# Amine-Selective Crosslinking of Collagen via Pre-Activated L-Glutamic Acid for Maintaining Ionic Interactions and Enhancing Mechanical and Biological Performance

**DOI:** 10.3390/polym18141766

**Published:** 2026-07-20

**Authors:** Senthilkumar Muthu, Seonae Kim, Jinsang Kim, Yongseon Wang, Inn Kyu Kang

**Affiliations:** 1 Research Institute, Ubiosis Co., Ltd., Jungwon-gu, Seongnam 13229, Republic of Korea; senthilmsknd@gmail.com (S.M.); jinsai@daum.net (J.K.); saingum@gmail.com (Y.W.); 2 Department of Orthopedic Surgery, College of Medicine, The Catholic University of Korea, Seoul 06591, Republic of Korea; kimseonae@catholic.ac.kr; 3 Department of Polymer Science and Engineering, Kyungpook National University, Daegu 41566, Republic of Korea

**Keywords:** type I collagen, cross-linking, viscoelasticity, tensile strength, MC3T3-E1 cells, cytotoxicity

## Abstract

Collagen-based biomaterials possess many advantages, such as low immunogenicity, biodegradability, biocompatibility, hydrophilicity, and ease of processability. Nevertheless, natural collagen has inherent limitations as an in vivo scaffold, including insufficient mechanical strength, low thermal stability, and low resistance to enzymatic degradation. To overcome these drawbacks, various approaches have been studied, such as mixing collagen with other biopolymers or inducing physical and chemical crosslinking. However, using non-biologically derived polymers or crosslinking agents carries the risk of persistence in the body, potentially causing cytotoxicity. Considering this, recent studies have reported that the molecular flexibility of collagen networks can be improved by activating the carboxyl groups of collagen chains using 1-ethyl-3-(3-dimethylaminopropyl) carbodiimide and N-hydroxysuccinimide and then crosslinking them through amide bonding with the amino groups present in the collagen chains, or by adding free L-lysine to induce a crosslinking reaction. When the carboxyl groups of collagen are activated and form covalent bonds with amino groups, native ionic interactions (e.g., salt bridges) may be reduced, which can potentially influence the stability of its inherent higher-order structure. In this study, we proposed a selective amine-targeted cross-linking strategy designed to minimize modification of collagen carboxyl groups while enhancing mechanical properties and cellular compatibility. First, free L-glutamic acid was pre-activated to cross-link collagen chains through amide bonds with the amino groups of L-lysine residues, thereby providing a cross-linking pathway intended to reduce the involvement of collagen carboxyl groups in the reaction. By controlling the concentration of L-glutamic acid, the cross-linking rate of the collagen could be controlled within a range of 10.26% to 25.02%. All cross-linked collagen scaffolds exhibited higher tensile strength compared to non-cross-linked scaffolds. Although the scaffolds with a high cross-linking rate (25.02%) displayed excellent mechanical properties, their cellular compatibility was relatively low. Conversely, collagen scaffolds with cross-linking rates of 10.26% and 14.43% demonstrated excellent mechanical properties and very high cellular compatibility, suggesting potential applications in the fields of biomedicine and tissue engineering. The present findings are consistent with the proposed selective cross-linking strategy; however, direct experimental verification of collagen carboxyl-group preservation will require complementary analytical studies.

## 1. Introduction

Collagen-based scaffolds have been widely utilized in biomedical applications, including artificial skin, bone, tendon, and cartilage, due to their excellent biocompatibility and biological functionality [1,2]. More recently, collagen has attracted increasing attention as a drug delivery system for controlled release applications. In such systems, collagen has been employed as a carrier for drugs and growth factors in various forms, including membranes, sponges, gels, microspheres, and injectable formulations [3]. As a principal structural protein of connective tissue, collagen plays a crucial role in physiological processes such as biological adaptation, wound healing, and tissue regeneration. Despite these advantages, collagen-based biomaterials still suffer from intrinsic limitations, including low thermal stability, insufficient mechanical strength, and poor resistance to enzymatic degradation, which restrict their broader application as in vivo scaffolds [4]. To overcome these drawbacks, numerous studies have focused on improving the physicochemical properties of collagen through cross-linking strategies. Cross-linking methods for collagen can generally be classified into physical, chemical, and biochemical approaches. For example, a scaffold with enhanced mechanical strength and water absorption capacity was fabricated by photo-crosslinking hyaluronic acid using tyrosine residues present in silk protein [5]. Physical cross-linking techniques such as ultraviolet (UV) irradiation, de-hydrothermal treatment (DHT), and gamma (γ) irradiation have also been applied to collagen materials [6,7,8]. Physical cross-linking methods offer advantages in that they can improve the physical and mechanical properties of collagen without introducing potentially toxic cross-linking agents or residual chemicals. However, these approaches often suffer from limitations, including difficulty in achieving a high and uniform degree of cross-linking and insufficient mechanical reinforcement when used alone [9]. Consequently, recent studies have reported that the properties of collagen-based materials can be further enhanced through synergistic effects achieved by combining physical cross-linking with additional cross-linking strategies [10]. In this context, the fabrication of collagen membranes with improved tensile strength and absorbability via physical cross-linking with poly-γ-glutamic acid has been reported [11].

Chemical cross-linking remains one of the most effective methods for enhancing the mechanical properties of collagen scaffolds. Commonly used chemical cross-linking agents include glutaraldehyde (GA), isocyanates, polyethylene glycol, polyepoxy compounds, inorganic cross-linkers, and 1-ethyl-3-(3-dimethylaminopropyl)carbodiimide (EDC) [12]. In addition, naturally derived cross-linkers such as genipin and tannic acid have been explored as alternatives [13,14]. However, synthetic cross-linkers such as aldehydes and isocyanates have been reported to exhibit potential cytotoxicity, raising concerns regarding their suitability for biomedical applications [15]. Although various chemical cross-linking agents have been developed, few are completely biocompatible and non-toxic. Recent studies have explored bio-derived cross-linkers, such as dopamine, to fabricate hydrogel patches based on hyaluronic acid or collagen [16]. EDC is widely regarded as a zero-length cross-linking agent capable of forming amide bonds between carboxyl and amino groups in polypeptide chains without leaving residual linker molecules [17]. Nevertheless, the limited inter-chain reactivity of EDC/NHS in collagen is primarily attributed to the restricted stereochemical accessibility of the reactive carboxyl and amino groups within the tightly packed triple-helical structure, which hinders effective formation of intermolecular crosslinks. [18,19]. This limitation may reduce cross-linking efficiency and, in some cases, lead to excessive stiffening or undesirable alterations in the collagen matrix. Usha et al. [19]. investigated the role of L-lysine in EDC/N-hydroxysuccinimide (NHS)-mediated cross-linking of collagen and reported that, in the absence of L-lysine, collagen exhibited a shear-dependent increase in viscosity (or shear-thickening behavior). In contrast, the presence of L-lysine significantly attenuated the increase in shear stress with shear rate, indicating enhanced molecular flexibility within the collagen network.

When collagen is treated with EDC/NHS, the carboxyl groups of the collagen chains are activated and can react not only with the ε-amino groups of the collagen’s L-lysine residues but also with the amino groups of externally added L-lysine [20]. As a result, both the carboxyl and amino groups of the collagen chains non-selectively participate in cross-linking reactions. In this case, there is a possibility that the intrinsic three-dimensional structure of collagen, which is formed mainly by ionic and hydrogen bonds, may be partially destroyed. To address this issue, the present study proposes a selective cross-linking strategy designed to preserve the intrinsic structure and biological activity of collagen. Specifically, the two carboxyl groups of L-glutamic acid (L-Glu) were pre-activated using EDC and NHS and subsequently introduced into a collagen solution. This approach was intended to induce cross-linking preferentially through the ε-amino groups of L-lysine residues exposed in the collagen chains, thereby minimizing direct activation of collagen carboxyl groups. Such a strategy was expected to better preserve the native molecular organization of collagen than conventional EDC/NHS cross-linking.

The primary objective of this study was to enhance the viscoelastic and mechanical properties of collagen gels while preserving their biological functionality. The selectivity of the pre-activated L-Glu approach relies on the following mechanistic rationale: Unlike conventional EDC/NHS-mediated crosslinking, where carboxyl groups on collagen (Asp/Glu residues) are directly activated, we first pre-activated the carboxyl groups of free L-glutamic acid using EDC/NHS to form a stable amine-reactive intermediate (NHS-ester). This pre-activated L-Glu then selectively reacts with the primary amine groups (mainly ε-amino groups of lysine residues) on collagen chains, forming amide bonds. Consequently, the carboxyl groups inherent to the collagen molecules are expected to remain largely unmodified, since the activated NHS esters are generated on free L-glutamic acid rather than on collagen itself. This strategy contrasts with standard zero-length crosslinking, which consumes both carboxyl and amine groups on collagen. To verify this, the viscoelastic behavior and mechanical strength of collagen cross-linked with L-Glu were evaluated using a rheometer and a rotational viscometer, respectively, and Fourier transform infrared spectroscopy (FT-IR) was performed to evaluate whether the characteristic spectral features associated with the collagen triple-helical structure were retained after the chemical cross-linking process. In addition, the biocompatibility of the crosslinked collagen was evaluated through cell viability and cytotoxicity tests using MC3T3-E1 cells.

## 2. Materials and Methods

### 2.1. Materials

Type I collagen was extracted from porcine skin tissue using the salt precipitation compression method, as previously reported [21]. Hydrochloric acid (first-grade reagent) was obtained from Deoksan Chemical (Daejeon, Republic of Korea). L-glutamic acid (L-Glu), 1-ethyl-3-(3-dimethylaminopropyl) carbodiimide (EDC), and N-hydroxysuccinimide (NHS) were purchased from Sigma-Aldrich Chemical Company (St. Louis, MO, USA). Dialysis membranes with a molecular weight cutoff (MWCO) of 10,000 Da, type I collagenase, and a hydroxyproline assay kit were also obtained from Sigma-Aldrich (St. Louis, MO, USA). Mouse pre-osteoblasts (MC3T3-E1) were purchased from the Korea Cell Bank (Seoul, Republic of Korea) and stored in liquid nitrogen until use. Cells were cultured in α-minimum essential medium (α-MEM; Gibco BRL, Grand Island, NY, USA) supplemented with 10% fetal bovine serum (FBS; Gibco) and 1.0% penicillin–streptomycin. Cell cultures were maintained at 37 °C in a humidified atmosphere containing 5% CO_2_, and the culture medium was replaced every two days. All other chemicals and solvents used in this study were of analytical grade and purchased from Sigma-Aldrich unless otherwise stated.

### 2.2. Preparation and Characterization of L-Glu-Cross-Linked Collagen Scaffolds

**Figure 1:**
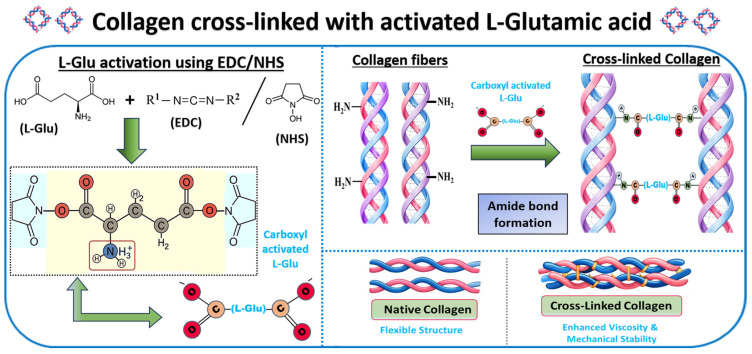
Schematic diagram of collagen cross-linking reaction using L-Glu. The two carboxyl groups of L-Glu were sequentially activated with EDC and NHS, and this was added to MES buffer containing type I collagen to produce a cross-linked collagen gel.

The high viscous collagen gel was prepared using activated L-Glu as a chemical cross-linking agent (Figure 1). The activation of L-Glu is carried out via the EDC-NHS catalyst. First, to prepare amino acid activation solutions of various concentrations, 0.4, 1.6, and 4 mM L-Glu were dissolved in 50 mL of 50 mM MES buffer (pH = 5.5). The α-amino group of L-glutamic acid has a pKa of approximately 9.6, allowing selective activation of its carboxyl groups under mildly acidic to neutral conditions. Then the catalyst EDC and the co-catalyst NHS were added sequentially (twice the amount of L-Glu concentration). After 3 h of activation of L-Glu, the solutions were mixed with collagen solution (final concentration of 1 wt.%) and the cross-linking reaction was carried out for 3 h with a gentle shaking at 4 °C. The obtained cross-linked collagen gels are termed as CCol-1 (collagen: L-Glu = 1.5 g: 0.4 mM), CCol-2 (collagen: L-Glu = 1.5 g: 1.6 mM and CCol-3 (collagen: L-Glu = 1.5 g: 4 mM). Afterwards, the collagen gels were transferred to a dialysis membrane after adjusting the pH to 6.4 using 100 mM Na_2_HPO_4_. Then the dialysis reaction was performed against distilled water at 4 °C for 24 h (the dialysate was replaced every 8 h). After the dialysis, the pH of the obtained cross-linked collagen gel is measured as 6.5. A control sample of non-cross-linked collagen gel (NCol) was prepared by adding type I collagen (1.5 g) to MES buffer (50 mM, pH = 5.5) without adding L-Glu activating solution, and then under reaction and dialysis conditions similar to those for cross-linked collagen gel. Afterwards, NCol and cross-linked collagen solutions (CCol-1, CCol-2, and CCol-3) were homogenized and transferred to culture dishes with diameters of 1.5 and 5 cm. The dishes were stored at −60 °C for 3 h to completely freeze the collagen solution and then lyophilized at −40 °C or lower. The obtained collagen scaffolds were separated from 24 wells and immersed in a double distilled water to examine swelling characteristics and shape retention. Furthermore, the cross-section and surface pore structure of the scaffolds were observed using a scanning electron microscope and other physico-chemical characterizations. Freeze-dried NCol and CCol samples (0.05 g) were each mixed with KBr (1 g) and finely ground using an agate mortar for 5 min. The resulting mixtures were compressed into transparent discs using a hydraulic press. Fourier transform infrared (FT-IR) spectra were recorded over the range of 4000–400 cm^−1^ using a Nicolet iS50 FT-IR spectrometer (Thermo Fisher Scientific, Waltham, MA, USA).

### 2.3. Rheological Characterization of L-Glu-Cross-Linked Collagen

Steady-shear viscosity measurements were performed using a Brookfield rotational viscometer (DV2TRV, Brookfield, Middleboro, MA, USA) equipped with an appropriate spindle selected based on the expected viscosity range of the collagen solution. The sample was loaded into the measurement container, ensuring complete immersion of the spindle without air entrapment. Viscosity was measured over a range of rotational speeds to evaluate shear-rate-dependent behavior. At each speed, the system was allowed to equilibrate until a stable torque reading was obtained. The apparent viscosity was calculated automatically by the instrument based on the measured torque, spindle geometry, and rotational speed. Viscoelastic properties were characterized using a rotational rheometer operated in oscillatory mode (ARES-G2, TA Inst., Seoul, Republic of Korea). A cone-and-plate geometry (cone-and-plate, 25 mm, 0.02 rad) was used, and the sample was carefully loaded to avoid structural disruption. A solvent trap was applied when necessary to prevent evaporation. Prior to frequency-dependent measurements, an oscillatory strain sweep was conducted at a fixed angular frequency to determine the linear viscoelastic region (LVR). Subsequent frequency sweep tests were then performed within the LVR, typically over an angular frequency range spanning several decades. The storage modulus (G′), loss modulus (G″), and complex viscosity were recorded as functions of frequency.

### 2.4. Tensile Testing of Collagen Scaffolds

A dialyzed collagen solution (pH 6.5, 1 wt.%) was dispensed 3 mL into 5 cm diameter polystyrene (PS) plates. The PS plates were frozen at −60 °C and then freeze-dried at low temperatures for 24 h to produce collagen scaffolds, which were used to measure their mechanical strength. The ends of the dumbbell-shaped collagen scaffolds were sandwiched between two sheets of fixing paper to simulate uniaxial tensile loading. A custom-made laser device was used to measure the cross-sectional area at least two locations. The approximate variation in the cross-sectional area of the scaffolds was within 10% of the average area. The ends of the collagen scaffolds were placed in a custom fixing device to secure them. After applying a preload of 0.005 N for 5 min, tensile testing was performed until failure using an Instron 5848 testing system (Instron Corp., Norwood, MA, USA) according to the standard protocol at a rate of 1 mm/min. The specimens were pulled at a rate of 1 mm/min to obtain stress–strain curves, and the tensile strength and elastic modulus were analyzed. For each mechanical value, the average of four specimens was used.

### 2.5. Enzymatic Degradation and Cross-Linking Degree Analysis

The enzymatic degradation behavior of the samples was analyzed by collagenase enzyme treatment. Non-cross-linked and cross-linked porous collagen scaffolds (70 mg) were immersed in phosphate-buffered saline (PBS, pH 7.4) containing 300 μg of type I collagenase (final volume sufficient to completely submerge the scaffolds). The samples were incubated at 37 °C for 12 h under gentle shaking to ensure uniform enzymatic degradation. After incubation, the enzymatic reaction was immediately quenched by placing the samples in ice water for 1 h [22]. The resulting collagen-digested solutions were transferred into centrifuge tubes and centrifuged at 10,000 rpm for 10 min. Visual observation showed that the non-cross-linked collagen scaffolds were completely degraded, resulting in a transparent solution without much precipitate (Figure S1a). On the other hand, as the enzymatic resistance percentage degree of crosslinking increased, the collagen scaffold formed a white precipitate due to its resistance to enzymatic degradation (Figure S1b–d). Both the supernatant and precipitate fractions were separately collected and transferred into glass test tubes. Subsequently, 6 M HCl solution was added (sufficient volume to fully hydrolyze the samples), and the mixtures were hydrolyzed in a preheated oil bath at 120 °C for 12 h to achieve complete amino acid hydrolysis. After hydrolysis, the samples were neutralized and subjected to hydroxyproline quantification using a chromogenic assay. The absorbance was measured at 558 nm, and the hydroxyproline content was calculated using a pre-established calibration curve. The degree of enzyme resistance was determined by comparing the amount of hydroxyproline released from non-cross-linked and cross-linked collagen samples, assuming that reduced hydroxyproline release corresponds to increased resistance to enzymatic degradation and thus a higher cross-linking degree [23].

The degree of cross-linking was analyzed by TNBS (trinitrobenzenesulfonic acid) colorimetric method featuring a high-volume acid hydrolysis step to precisely characterize the cross-linking degree (CD%) of highly viscous type I collagen hydrogels. The estimation of free amino group density after the TNBS assay treatment and acid hydrolysis of the native and cross-linked collagen, the degree of cross-linking was calculated. To establish a high-precision reference model for absolute primary amine quantitation, L-glycine was used as a primary amine quantification by preparing the standard calibration graph of free amine in the range of 0 μg to 100 μg using 0.1 M bicarbonate (NaHCO_3_) buffer solution.

For each sample (NCol, CCol-1,2-3), high-precision massing was conducted with 20.0 mg (0.0200 g) of the respective 1 wt.% hydrogel directly onto the bottom center of the vial. Subsequently, 1000 µL of 0.1 M sodium bicarbonate buffer (pH 8.50) was introduced into all sample vials. After that 100 µL of a 10 wt.% sodium dodecyl sulfate (SDS) surfactant solution was pipetted and vigorously vortexed for 10 s and incubated at 40 °C for 1 h. This surfactant-driven thermal treatment disrupted non-covalent structural loops and uncoiled the rigid collagen triple-helices, ensuring that 100% of the sterically hidden amino groups of the constituent lysine residues were exposed to the aqueous phase. After that, 500 µL of 0.1 wt.% TNBS (2,4,6-trinitrobenzenesulfonic acid) working solution was introduced. Following brief vortexing, all the vials were hermetically sealed, mounted on a rack, and wrapped securely in a double layer of aluminum foil to guarantee absolute darkness.

The light-shielded rack was then incubated at 37 °C for 1 h to allow the TNBS to covalently label the free primary amines via a nucleophilic aromatic substitution cascade. To freeze the reaction kinetics, acid hydrolysis step was performed using 2.0 mL of 6 M HCl and heat treatment at 105 °C for 1 h. The strong acid instantly decreased the solution pH below 2.0, protonating all unreacted primary amines into unreactive ammonium ions (NH_3_^+^) and permanently halting the trinitrophenylation reaction. Similar experimental procedure was carried out for the standard glycine samples. Finally, 200 µL of each acid hydrolyzed samples were transferred into individual wells of a clean, UV-transparent, flat-bottom 96-well microplate. The microplate was loaded into a spectrophotometer reader, and the absolute optical absorbance was measured at a detection wavelength of 345 nm.

A linear calibration curve was plotted (Net A_sample_) vs. Glycine Mass), and the linear regression parameters (Slope (m), Y-intercept (C), and (R^2^) were computed. The absolute mass of free primary amines (expressed as micrograms of glycine equivalents) for each collagen replicate was interpolated using the standard curve equation:

$$Amine\;mass\;\left(M\right)=\;\frac{Net\;A_{sample}\;-C}{m}$$

The cross-linking degree (CD%) for the CCol variants was determined relative to the native collagen control sample via the equation:

$$Degree\;of\;crosslinking\;\left(\%\right)=\left(1-\;\frac{\;M_{CCol}\;}{\;M_{NCol}}\;\right)\times 100$$

### 2.6. In Vitro Biological Evaluation

#### 2.6.1. Preparation of Extracts

To evaluate the biological effects of the collagen scaffold, extracts were prepared using an indirect contact method. Each substrate (NCol, CCol-1, CCol-2, and CCol-3) was immersed in 4 mL of α-MEM (A10490-01, Gibco, New York, NY, USA) supplemented with 10% FBS at a weight of 0.8 g and extracted for 72 h at 37 °C. Following the extraction, the samples were centrifuged at 2000 rpm for 10 min to collect the supernatant. The prepared extracts were stored at 4 °C until further use in subsequent experiments.

#### 2.6.2. Cytotoxicity

MC3T3-E1 cells were seeded in 24-well plates at a density of 2 × 10^4^ cells/mL using α-MEM supplemented with 10% FBS and stabilized for 24 h. The culture medium was replaced with the prepared collagen scaffold extracts. After 72 h of incubation, cell cytotoxicity was characterized using calcein acetoxymethyl ester (calcein-AM) and ethidium homodimer-I (EthD-I) dyes (L3224, Thermo Fisher Scientific, Waltham, MA, USA) [24]. The cells were washed with PBS and stained with a solution of 2 μM calcein-AM and 4 μM EthD-I for 30 min in the dark at room temperature. Subsequently, the stained cells were visualized using a fluorescence microscope (IX71, Olympus, Tokyo, Japan), where live and dead cells appeared green and red, respectively.

#### 2.6.3. Cell Proliferation

MC3T3-E1 cells were suspended in α-MEM supplemented with 10% FBS and seeded into 96-well plates at densities of 1 × 10^4^, 2 × 10^4^ cells/well (100 μL/well). Following a 24 h incubation for cell attachment, the culture medium was replaced with the prepared collagen scaffold extracts. At 24 and 72 h post-treatment, 10 μL of WST-1 (Roche, Basel, Switzerland) solution was added to each well (1:10 final dilution) and incubated for 3 h at 37 °C under 5% CO_2_ in the dark [25]. Culture medium containing 10% WST-1 without cells served as a blank control. The absorbance was measured at 450 nm using a microplate reader (SoftMax i3x, Molecular Devices, San Jose, CA, USA).

## 3. Results and Discussion

### 3.1. Characterization of L-Glu-Cross-Linked Collagen Scaffolds

**Figure 2:**
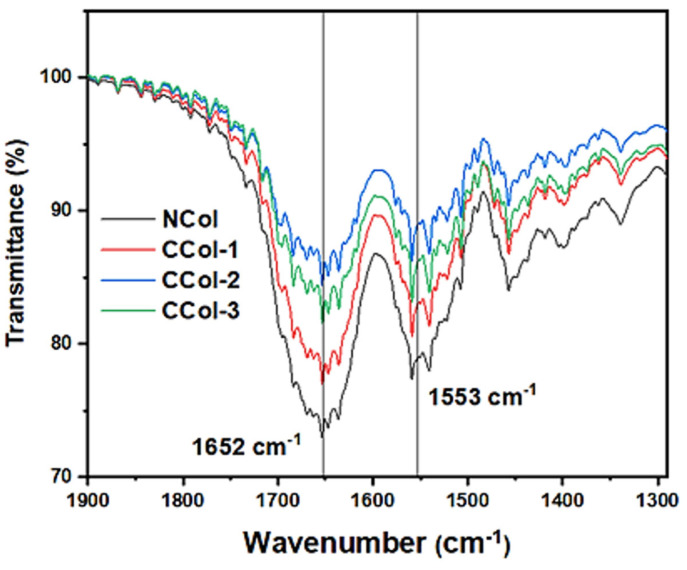
FT−IR spectra of natural collagen (NCol) and L-Glu-cross-linked collagen (CCol series). Samples were prepared by the KBr method, and the spectrum amplitude was narrowed and magnified to 1300–1900 cm^−1^.

To investigate whether L-Glu cross-linking alters the characteristic FT-IR features associated with the collagen triple-helical structure, FT-IR spectra of non-cross-linked and cross-linked collagen were analyzed (Figure 2 and Figure S2). Typically, retention of the characteristic amide I and amide II bands is considered consistent with preservation of the collagen triple-helical structure, although FT-IR alone does not provide direct structural confirmation appearing around 1650 cm^−1^ and 1550 cm^−1^, respectively. As shown in Figure 2, both uncross-linked and cross-linked collagen exhibited amide I and II peaks at 1652 cm^−1^ and 1553 cm^−1^ without noticeable shifts, suggesting that no apparent disruption of the characteristic triple-helical structure occurred following L-Glu cross-linking, based on FT-IR analysis. In contrast, the amide A band (3300–3500 cm^−1^), shown in Figure S2, exhibited a gradual decrease in intensity with increasing crosslinker concentration [26]. This reduction can be attributed to the consumption of free amine groups (–NH_2_) during the crosslinking reaction and possible rearrangement of hydrogen bonding interactions.

In this study, we aimed to improve mechanical reinforcement and cellular compatibility by selectively targeting amine groups for cross-linking while preserving collagen carboxyl groups, thereby potentially maintaining native ionic interactions. The FT-IR results suggest that this strategy does not cause detectable disruption of the characteristic triple-helical structure. Overall, these FT-IR results suggest that L-Glu-mediated cross-linking modifies the local chemical environment of collagen without causing detectable changes in the characteristic spectral features associated with the triple-helical structure within the studied concentration range. It should be noted that FT-IR provides indirect information on collagen molecular structure. Therefore, while the present FT-IR results are consistent with retention of the characteristic collagen structure after cross-linking, definitive confirmation of triple-helical preservation requires complementary techniques such as circular dichroism (CD) spectroscopy or X-ray diffraction.

**Figure 3:**
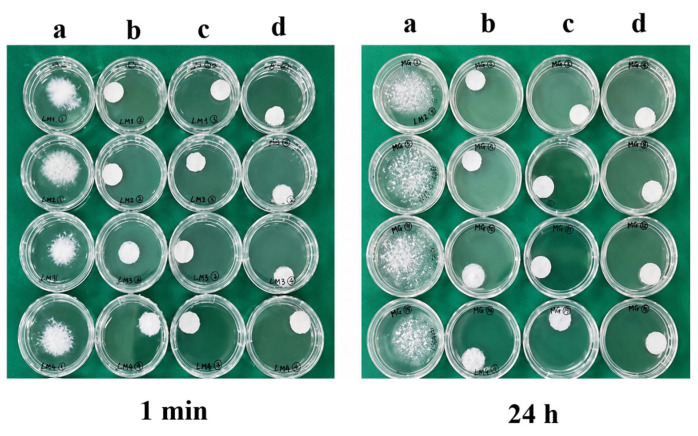
Swelling behavior of freeze-dried collagen scaffolds prepared from dialyzed native collagen (NCol) and L-Glu-cross-linked collagen solutions after immersion in distilled water for 1 min and 24 h. The labels a–d indicate the sample identities within each photograph: (a) NCol, (b) CCol-1, (c) CCol-2, and (d) CCol-3. The left and right photographs represent the scaffolds after 1 min and 24 h of immersion, respectively.

To evaluate the structural stability of collagen networks in aqueous environments, the swelling behavior of scaffolds prepared with and without L-Glu crosslinking was examined (Figure 3). The non-cross-linked collagen scaffold (NCol) exhibited rapid and extensive swelling upon immersion in distilled water. It absorbed a large amount of water and significantly increased in volume within a minute. After 24 h, the scaffold lost its original shape and became dispersed in the solution, indicating severe structural collapse. This behavior reflects the intrinsic hydrophilicity of collagen and the lack of sufficient intermolecular interactions to maintain structural integrity under hydrated conditions [27]. In contrast, the L-Glu-cross-linked scaffolds (CCol-1, CCol-2, and CCol-3) showed markedly improved water stability. All cross-linked samples exhibited minimal swelling after 1 min and maintained their overall shape even after 24 h of immersion. This enhanced stability can be attributed to the formation of intermolecular cross-links, which stabilize the collagen network and limit structural deformation in aqueous environments. Notably, increasing the L-Glu concentration from 0.4 mM to 4.0 mM did not result in significant differences in macroscopic swelling behavior. This suggests that a relatively low crosslinker concentration is sufficient to achieve a critical crosslinking density required for structural stabilization [20].

**Figure 4:**
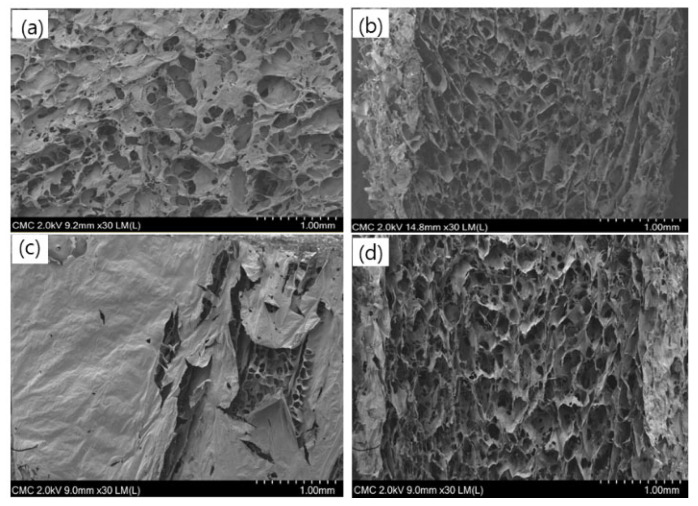
Scanning electron microscopy (SEM) images of collagen scaffolds: (**a**) surface of cross-linked collagen, (**b**) cross-section of cross-linked collagen, (**c**) surface of non-cross-linked collagen, and (**d**) cross-section of non-cross-linked collagen scaffolds.

Figure 4 shows the surface and cross-sectional morphologies of collagen scaffolds observed via SEM, highlighting the significant influence of cross-linking on microstructural features. The cross-linked collagen scaffold exhibited a rough and heterogeneous surface with irregularly distributed pores (Figure 4a), indicating that cross-linking disrupted the regular packing of collagen fibrils and inhibited surface densification during freeze-drying. This effect is attributed to the formation of intermolecular covalent bonds that restrict chain mobility. In the cross-section (Figure 4b), a well-defined three-dimensional porous network with interconnected pores (100~400 μm) was observed, suggesting that cross-linking stabilizes the collagen matrix during ice templating and sublimation. Such an interconnected architecture is beneficial for mass transport and cellular infiltration in tissue engineering applications [28]. In contrast, the non-cross-linked scaffold showed the formation of a dense, nonporous surface layer (Figure 4c), likely resulting from fibril collapse or rearrangement in the absence of structural stabilization. Although the cross-section (Figure 4d) still displayed a porous structure with pore sizes ranging from 100 to 500 μm, the distribution was broader and less uniform, indicating reduced structural stability during freeze-drying. Overall, cross-linking suppresses surface densification while promoting a stable and interconnected pore network with improved uniformity, which is expected to enhance the mechanical integrity and biological performance of collagen-based scaffolds.

### 3.2. Rheological Characterization of L-Glu-Cross-Linked Collagen

**Figure 5:**
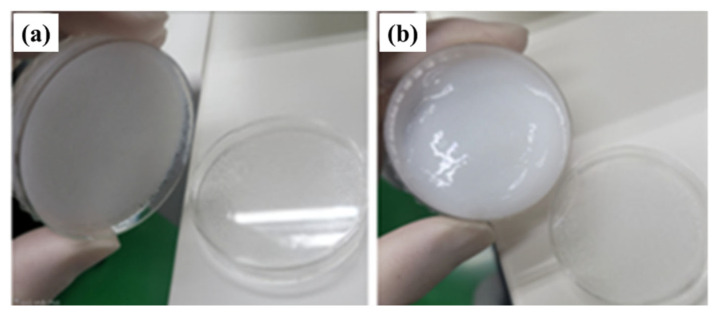
After activating L-Glu (1.6 mM) with EDC/NHS, it was mixed with type I collagen (1.5 g) and reacted with stirring in MES buffer (pH 5.5) at 4 °C for 24 h ((**a**) CCol-2). Meanwhile, for the non-cross-linked collagen solution, type I collagen (1.5 g) was dissolved in MES buffer (pH 5.5) and stirred at 4 °C for 24 h ((**b**) NCol).

Figure 5 presents the macroscopic appearance of 1 wt.% type I collagen solutions after 24 h of reaction in the presence and absence of L-Glu. A clear difference in flow behavior was observed between the cross-linked and non-cross-linked samples. When the culture dish containing the L-Glu-cross-linked collagen (CCol-2) was tilted, the material exhibited little or no flow (Figure 5a). In contrast, the non-cross-linked collagen solution (NCol) flowed readily under the same conditions (Figure 5b). This simple tilting test qualitatively demonstrates that chemical cross-linking significantly alters the rheological behavior of the collagen system, transforming it from a fluid-like solution into a viscoelastic or weak gel. In acidic aqueous environments, collagen molecules generally exist as triple-helical structures that interact through relatively weak physical forces such as hydrogen bonding, electrostatic interactions, and physical chain entanglement. At relatively low concentrations (e.g., 1 wt.%), these interactions are insufficient to suppress macroscopic flow, resulting in solution-like behavior as observed for the non-cross-linked sample (Figure 5b) [29]. On the other hand, the cross-linking reaction by L-glutamic acid promotes intermolecular interactions between collagen chains, effectively connecting adjacent molecules and creating a three-dimensional network structure. The formation of this interconnected network restricts molecular mobility and increases resistance to gravitational deformation, thereby producing the observed gel-like behavior (Figure 5a) [30]. The absence of flow in the cross-linked sample suggests that the collagen system undergoes a transition from a viscous liquid to a viscoelastic solid-like material. Such behavior is consistent with the formation of a percolated molecular network that distributes mechanical stress throughout the system [31,32]. Cross-linking therefore increases the effective connectivity among collagen molecules and enhances structural integrity, which is an important characteristic for biomedical materials requiring shape retention and mechanical stability.

As shown in Table 1, the viscosity of type I collagen solutions was strongly influenced by the concentration of the cross-linking agent, L-Glu. The native collagen solution (NCol) exhibited an average viscosity of 114 mPa.s, which reflects the intrinsic rheological behavior of collagen governed primarily by intermolecular interactions and physical chain entanglement in the absence of chemical cross-linking. All L-Glu-cross-linked samples (CCol series) showed higher viscosities than NCol, indicating that L-Glu effectively promoted intermolecular interactions and network formation within the collagen system.

At a low L-Glu concentration of 0.4 mM (CCol-1), the viscosity increased to 140 mPa.s, corresponding to approximately a 1.2-fold increase compared with NCol. This moderate increase suggests the initial formation of cross-links between collagen molecules, which restrict molecular mobility and increase resistance to flow. When the L-Glu concentration was increased to 1.6 mM (CCol-2), the viscosity rose markedly to 263 mPa.s, representing a 2.3-fold increase relative to NCol. This pronounced enhancement indicates the formation of a more developed three-dimensional network structure due to increased cross-linking density. The higher connectivity among collagen chains likely intensified molecular entanglement and structural integrity, resulting in a substantial increase in resistance to shear deformation [33]. These results suggest that 1.6 mM L-Glu provides an optimal condition within the tested range for efficient network formation in the collagen solution. Interestingly, further increasing the L-Glu concentration to 4.0 mM (CCol-3) resulted in a decrease in viscosity to 186 mPa.s, although this value remained higher than that of NCol. This non-monotonic behavior suggests that excessive cross-linker concentration does not necessarily lead to continuous enhancement of viscosity. Over-cross-linking may induce localized aggregation or structural heterogeneity, which can disrupt uniform network formation and reduce effective intermolecular connectivity.

To investigate the rheological properties of cross-linked collagen samples, which exhibits the highest viscosity, in more detail, viscoelastic properties were evaluated through dynamic mechanical analysis, and the results are shown in Table 2 with the comparison of pristine collagen. The storage modulus (G′), representing the elastic component, and the loss modulus (G″), representing the viscous component, provide insight into the mechanical behavior of the samples. In general, when G′ exceeds G″, the material exhibits solid-like (gel-like) behavior, whereas when G″ exceeds G′, the material behaves more like a viscous liquid (sol) [34]. All the NCol and CCol-1, 2, 3 samples exhibited higher storage modulus values than loss modulus values (NCol: G′ = 20.9 Pa, G″ = 7.27 Pa; CCol-1: G′ = 56.2 Pa, G″ = 17.3 Pa, CCol-2: G′ = 75.1 Pa, G″ = 25.1 Pa and CCol-3: G′ = 68.3 Pa, G″ = 23.4 Pa), indicating that the samples predominantly exhibit gel-like viscoelastic behavior. Notably, the storage modulus of CCol-2 was higher than that of NCol, suggesting that cross-linking with L-Glu enhances the elastic network structure of the collagen matrix. The loss tangent (tan δ = G″/G′) further reflects the relative contribution of viscous and elastic responses. The tan δ value of NCol was 0.35, whereas that of cross-linked collagen sample exhibits decreased tan δ of 0.33. A lower tan δ value indicates a more elastic, solid-like material [35]. Therefore, the reduced tan δ observed for cross-linked samples confirms that L-Glu-mediated cross-linking strengthens the collagen network and enhances gel-like mechanical behavior.

Collectively, these results demonstrate that the concentration of the crosslinking agent L-glutamic acid (L-Glu) plays a crucial role in controlling the viscosity and viscoelastic properties of collagen solutions. An optimal crosslinking agent concentration (1.6 mM in this study) exists where intermolecular connectivity and network formation are maximized, thereby enhancing rheological performance.

However, excessive crosslinking agent concentrations can lead to structural non-uniformity and reduced rheological efficiency. These findings highlight the importance of controlling crosslinking agent concentration in the development of collagen-based biomaterials, as rheological properties directly influence processability, structural stability, and potential biomedical applications.

### 3.3. Tensile Mechanical Properties of L-Glu-Cross-Linked Collagen Scaffolds

**Figure 6:**
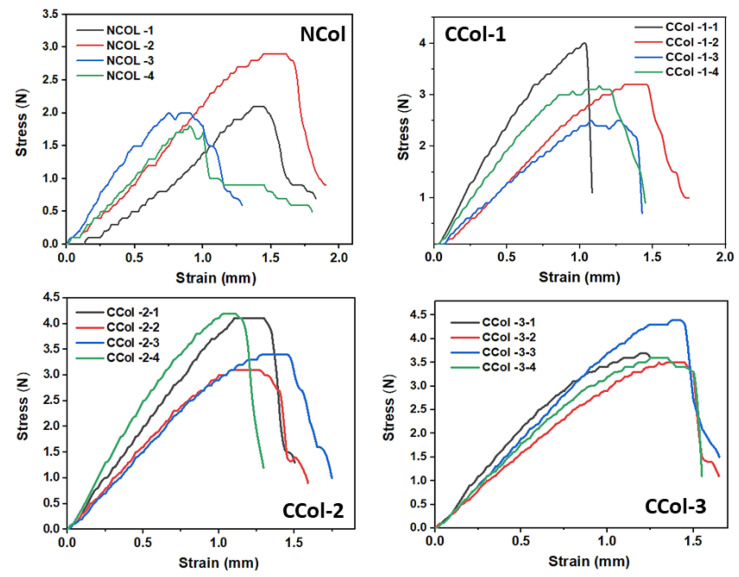
Stress–strain curves of non-cross-linked collagen (NCol) and L-Glu-cross-linked collagen scaffolds (CCol series).

The tensile mechanical properties of collagen scaffolds with varying degrees of L-Glu-mediated cross-linking were evaluated using stress–strain analysis (Figure 6), and the corresponding tensile loads are summarized in Table 3. The non-cross-linked scaffold (NCol) exhibited a relatively low tensile load (2.4 N) and tensile strength (0.0062 MPa), reflecting the limited mechanical stability of physically assembled collagen networks dominated by weak intermolecular interactions such as hydrogen bonding. In contrast, L-Glu-cross-linked scaffolds (CCol series) showed markedly improved mechanical performance, with average tensile loads increasing to 3.13 N (CCol-1), 3.72 N (CCol-2), and 3.76 N (CCol-3), corresponding to approximately 1.4- ~ 1.7-fold enhancement compared to NCol. Similarly, the tensile strength of the cross-linked collagen scaffolds enhanced to 0.089 MPa, 0.1 MPa and 0.11 MPa, respectively for CCol-1, CCol-2 and CCol-3.

The elastic modulus values were also estimated from the stress–strain curve and denoted in Table 3. The NCol scaffold exhibits an elastic modulus of 2.18 MPa, which is the intrinsic collagen inter-fibrillar structural characteristics of the non-cross-linked collagen. The cross-linked collagen samples exhibit linear enhancement in elastic modulus such as 2.86, 3.85 and 3.90 MPa, respectively, for the CCol-1, CCol-2 and CCol-3 samples. These results provide further evidence that L-Glu-mediated cross-linking enhances the resistance of collagen scaffolds to deformation and increases their load-bearing capacity, suggesting the formation of a mechanically reinforced network. These improvements are consistent with the rheological behavior of the precursor solutions.

The viscosity increased from 114 mPa.s for NCol to a maximum of 263 mPa.s for CCol-2, suggesting enhanced intermolecular interactions and network formation in the presence of L-Glu. Such increases in viscosity imply reduced chain mobility and greater molecular connectivity, which are effectively retained during freeze-drying and contribute to the mechanical reinforcement of the resulting porous scaffolds. Notably, CCol-2 exhibited both the highest viscosity and one of the highest tensile strengths, indicating the presence of an optimal cross-linking density that maximizes load-bearing efficiency while maintaining structural homogeneity [36]. In contrast, a slight decrease in viscosity at higher cross-linker concentration (CCol-3), despite sustained mechanical improvement, suggests that excessive cross-linking may induce structural heterogeneity or localized aggregation, limiting further enhancement in macroscopic properties. Overall, L-Glu-mediated cross-linking significantly improves the tensile properties of collagen scaffolds by promoting intermolecular network formation, and the viscosity of precursor solutions serves as a useful indicator for predicting the mechanical performance of the resulting materials.

### 3.4. Quantitative Evaluation of Enzyme Resistance Behavior of Cross-Linked Collagen and Analysis of Degree of Cross-Linking by TNBS Assay

The cross-linked and pristine collagen samples were first treated with collagenase to enzymatically decompose the non-cross-linked portions of the collagen chains. The undigested collagen chains were removed by high-speed centrifugation, and the supernatant was hydrolyzed with hydrochloric acid to release the hydroxyproline residue. The amount of released Hyp was quantified by measuring the absorbance at 558 nm and converting the value to concentration using the calibration curve [37]. The standard calibration curve showing the relationship between the hydroxyproline (Hyp) concentration (*x*-axis) and the absorbance at 558 nm (*y*-axis) after reaction with a chromogenic reagent. A strong linear correlation was observed within the tested concentration range (R^2^ = 0.9769), indicating that the absorbance at 558 nm is proportional to the Hyp concentration. The same analytical procedure was applied to non-cross-linked collagen samples as a control. By comparing the Hyp concentration released from cross-linked collagen with that from non-cross-linked collagen, the enzyme resistance percentage through cross-linking was calculated. The calculated values are summarized in Table 4.

As shown in Table 4, the enzyme resistance percentage of cross-linking increased proportionally with the concentration of the cross-linking agent (L-Glu), indicating that higher L-Glu concentrations promote more extensive collagen cross-link formation. In this study, non-cross-linked collagen (NCol) and cross-linked collagen (CCol) samples were subjected to enzymatic hydrolysis using collagenase, followed by centrifugation to separate soluble degradation products from any insoluble residues. Visual inspection (Figure S1a) confirmed that non-cross-linked collagen (NCol) was fully degraded by collagenase, resulting in complete solubilization with not much sediment formation. On the other hand, the cross-linked collagen samples (Figure S1c,d) produced visible precipitates after collagenase treatment as the degree of cross-linking increased to some extent. This sediment corresponds to the enzyme-resistant, cross-linked collagen domains, as collagenase primarily cleaves the triple-helical regions of non-cross-linked or loosely associated collagen chains but does not efficiently recognize or degrade highly cross-linked portions where covalent bridges sterically hinder or alter substrate recognition [38]. As a result, collagenase digestion selectively breaks down natural collagen to release amino acids including hydroxyproline (Hyp), while the cross-linked sites are not broken down and remain in an insoluble state, precipitating when subjected to high-speed centrifugation [22,39]. The enzyme resistance percentage of crosslinking is therefore quantifiable by comparing the Hyp concentration in the supernatant from cross-linked samples to that from non-cross-linked collagen (NCol, set as 100% release). Lower Hyp release in the supernatant indicates a higher proportion of cross-linked collagen that resists enzymatic degradation.

As shown in Table 4, the amount of hydroxyproline (Hyp) released from collagen progressively decreased with increasing concentrations of the crosslinking agent L-Glu, indicating enhanced crosslinking within the collagen matrix. For CCol-1 prepared with 0.4 mM L-Glu, 96.5% of Hyp was released relative to non-crosslinked collagen (NCol), corresponding to a low enzyme resistance percentage of 3.5%. When the L-Glu concentration was increased to 1.6 mM (CCol-2), the relative Hyp release decreased to 87.6%, yielding an enzyme resistance percentage of 12.4%. A further increase to 4.0 mM L-Glu (CCol-3) significantly reduced the Hyp release to 63.3%, corresponding to the enzyme resistance percentage of 36.7%. These results demonstrate a clear inverse relationship between L-Glu concentration and Hyp release, reflecting a dose-dependent increase in crosslinking efficiency. The degree of enzyme resistance was estimated according to the following equation:

Degree of enzyme resistance (%) = 100 − relative Hyp release (%)

The quantitative tracking of free amine densities across the hydrogel cohorts revealed a clear, dose-dependent staircase reduction directly mirroring the feed concentration of the activated L-glutamic acid spacers. From the analysis of free amine concentration from the TNBS assay of the native collagen matrix control (NCol) exhibited an average net absorbance of 0.0191, which interpolated to a baseline free primary amine mass of 6.65 µg (glycine equivalents). This reading represents the maximum unhindered state of the protein’s native lysine residues. Upon introduction of the cross-linking mixtures, the net optical densities stepped downward in tandem with increasing spacer density, tracking at 0.0175 for CCol-1 (0.4 mM), 0.01685 for CCol-2 (1.6 mM), and 0.0152 for CCol-3 (4.0 mM). When mapped onto the calibration equation, the remaining free amine masses dropped systematically to 5.96 µg, 5.68 µg, and 4.98 µg, respectively. Applying the mass-reduction equation, the final cross-linking degree (CD%) were determined to be 10.26% for CCol-1, 14.43% for CCol-2, and 25.02% for CCol-3. This steady upward climb confirms that our formulation parameters successfully controlled the internal covalent network density of the biomaterials [40,41]. The calculated values are summarized in Table 5.

This enzyme-based assay provides a practical indirect measure of crosslinking extent, complementing other characterization methods such as enzymatic weight loss, swelling analysis, or quantification of free amine groups. Overall, the results confirm that the crosslinking degree of collagen–L-Glu systems can be effectively controlled by adjusting the concentration of L-Glu, enabling tunable enzymatic stability and structural integrity of collagen-based biomaterials.

### 3.5. Effect of Cross-Linking on Osteoblast Viability and Proliferation

**Figure 7:**
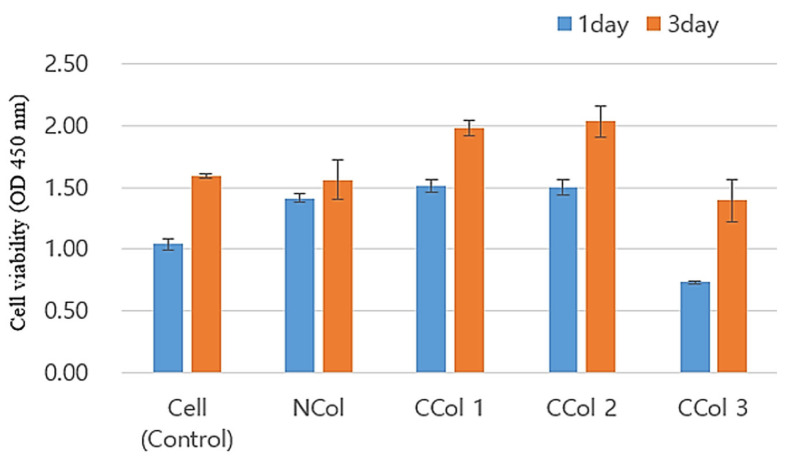
WST-1 assay results showing metabolic activity of osteoblasts cultured on collagen scaffolds for 1 and 3 days. Data are presented as mean ± SD (*n* = 3). Statistical significance was determined relative to NCol (*p* < 0.05).

The cytocompatibility of collagen scaffolds with varying cross-linking densities was assessed using live/dead staining (Figure S3) and WST-1 assays (Figure 7). After 24 h of culture, osteoblasts on all L-Glu-cross-linked collagen scaffolds (CCol series) exhibited strong green fluorescence similar to natural collagen scaffolds (NCol), with almost no red signal. This indicates high cell viability and confirms that L-Glu-mediated cross-linking does not introduce cytotoxic residues. In contrast to conventional cross-linkers such as glutaraldehyde, which may leave cytotoxic aldehyde groups, the present system preserves the intrinsic biocompatibility of collagen [42]. The WST-1 assay results further revealed a cross-linking-dependent cellular response. At day 1, NCol and moderately cross-linked scaffolds (CCol-1 and CCol-2) showed comparable metabolic activity, whereas the highly cross-linked sample (CCol-3) exhibited slightly reduced activity. By day 3, CCol-1 and CCol-2 demonstrated significantly higher metabolic activity than NCol (*p* < 0.05), indicating enhanced osteoblast proliferation. This improvement is attributed to the increased structural stability and mechanical integrity of the scaffolds, which provide a more favorable microenvironment for cell attachment and growth [28,36]. In contrast, CCol-3, despite maintaining high cell viability, showed relatively lower metabolic activity at day 3, suggesting that excessive cross-linking may hinder cell proliferation. This effect is likely associated with reduced matrix flexibility, limited pore accessibility, or restricted nutrient transport within an overly dense network. Overall, L-Glu-mediated cross-linking enables the tuning of scaffold properties without compromising cytocompatibility. Moderate cross-linking enhances cellular activity, whereas excessive cross-linking may limit proliferation, highlighting the importance of optimizing cross-linking density to balance mechanical reinforcement with biological performance.

## 4. Conclusions

In this study, the carboxyl groups of L-glutamic acid were pre-activated to preferentially react with the ε-amino groups of collagen. This strategy was intended to minimize modification of collagen carboxyl groups while promoting selective cross-linking. This selective cross-linking strategy was designed to minimize modification of collagen carboxyl groups and to better retain the native molecular organization of collagen. FT-IR analysis suggested that the characteristic spectral features associated with the collagen triple-helical structure were retained after cross-linking. The resulting L-Glu-cross-linked collagen was systematically evaluated in terms of its rheological, physicochemical, structural, enzymatic, and biological properties. Chemical cross-linking significantly altered the rheological behavior of collagen solutions. While non-cross-linked collagen exhibited low viscosity and solution-like characteristics, L-Glu-cross-linked collagen formed high viscoelastic, gel-like structures with reduced flowability. This transition is attributed to the formation of intermolecular networks that restrict molecular mobility and enhance resistance to deformation. Cross-linking also markedly improved the stability of collagen in aqueous environments. Non-cross-linked collagen rapidly swelled and dissolved, while L-Glu-cross-linked scaffolds maintained structural integrity with minimal swelling, even after prolonged immersion. This enhanced stability is likely due to increased cross-link density, which limits polymer chain relaxation and reduces water penetration. Hydroxyproline analysis confirmed that the degree of cross-linking increased with L-Glu concentration. Accordingly, cross-linked collagen exhibited significantly greater resistance to collagenase digestion, indicating the formation of enzyme-resistant domains and modification of the higher-order structure. Scanning electron microscopy revealed that cross-linking influenced scaffold microstructure, resulting in rough surfaces and well-interconnected porous networks while suppressing the formation of dense surface layers. These structural features are beneficial for tissue engineering, as they promote cell infiltration and efficient mass transport. Importantly, cytocompatibility analysis using live/dead staining demonstrated high cell viability in osteoblasts cultured on both non-cross-linked and cross-linked scaffolds, with no detectable cytotoxic effects. This suggests that L-Glu-mediated cross-linking does not produce harmful residues, in contrast to conventional cross-linkers such as glutaraldehyde. As a naturally occurring amino acid, L-Glu serves as a biocompatible cross-linking agent that enhances collagen stability without compromising biological safety.

Overall, L-Glu-mediated cross-linking provides a mild and effective approach to modulate the rheological, structural, and biological properties of type I collagen. Based on FT-IR analysis, this strategy appears to retain the characteristic spectral features of the collagen triple-helical structure while improving physiological characteristics and cytocompatibility. The resulting materials exhibit improved viscosity, aqueous stability, enzymatic resistance, and cytocompatibility, highlighting their potential for biomedical applications, including injectable hydrogels and tissue engineering scaffolds for cartilage and tendon regeneration. Although the present results are consistent with the proposed selective cross-linking strategy, direct verification of collagen carboxyl-group preservation and triple-helical conformation will require complementary analytical techniques in future studies.

## Figures and Tables

**Table 1:** Changes in the viscosity of collagen solutions according to the concentration of cross-linking agent (L-Glu).

Sample	Ratio		Viscosity (mPa.s)				
	Collagen (g)	L-Glu (mg)	n1	n2	n3	n4	Average
NCol	1.5	0	119	119	104	114	114
CCol-1	1.5	2.9	139	134	134	150	140
CCol-2	1.5	11.8	222	300	248	279	263
CCol-3	1.5	29.4	196	170	201	165	186

n: Number of measurements, collagen concentration: 1 wt.%, pH: 6.5, rotation speed: 10 rpm, temperature of rotary stage: 25 °C.

**Table 2:** Viscoelastic properties of 1 wt.% native collagen and L-Glu-cross-linked collagen solutions.

Sample	Storage Modulus (G′) Pa	Loss Modulus (G″) Pa	Tan δ (G′/G″)	Complex Viscosity (η)
NCol	20.9	7.27	0.35	3.53
CCol-1	56.2	17.3	0.31	9.36
CCol-2	75.1	25.1	0.33	12.6
CCol-3	68.3	23.4	0.34	11.5

**Table 3:** Tensile load and tensile strength of non-cross-linked collagen (NCol) and L-Glu-cross-linked collagen scaffolds (CCol) calculated from the stress–strain curves.

Sample	Tensile Load (N)					Tensile Strength (MPa)	Elastic Modulus (MPa)
	n1	n2	n3	n4	Average		
NCol	2.1	2.9	2.8	1.8	2.4	0.062	2.18
CCol-1	4.0	2.8	2.5	3.1	3.13	0.089	2.86
CCol-2	4.1	3.1	3.4	4.2	3.72	0.1	3.85
CCol-3	3.7	3.4	4.3	3.6	3.76	0.11	3.90

**Table 4:** Relationship between L-glutamic acid concentration and Hyp. Concentration released from collagen.

Sample	Ratio		Hyp. Released (μg/μL)	Relative Amount of Hyp. Released (%)	Enzyme Resistance (%)
	Collagen (g)	L-Glu (mg)			
NCol	1.5	0	0.437	100	0
CCol-1	1.5	2.9	0.422	96.5	3.5
CCol-2	1.5	11.8	0.383	87.6	12.4
CCol-3	1.5	29.4	0.277	63.3	36.7

**Table 5:** TNBS analysis of primary free amino group density and corresponding structural cross-linking degree (CD%) of cross-linked collagen samples.

Sample	Ratio		Net Optical Density at 345 nm	Estimated Free Amine Content (μg)	Cross-Linking Degree (CD %)
	Collagen (g)	L-Glu (mg)			
NCol	1.5	0	0.0191	6.645	0
CCol-1	1.5	2.9	0.0175	5.963	10.26
CCol-2	1.5	11.8	0.01685	5.686	14.43
CCol-3	1.5	29.4	0.0152	4.983	25.02

## Data Availability

The original contributions presented in this study are included in the article/Supplementary Materials. Further inquiries can be directed to the corresponding author.

## Supplementary Materials

The following supporting information can be downloaded at: https://www.mdpi.com/article/10.3390/polym18141766/s1. Figure S1: Non-cross-linked collagen and cross-linked collagen (70 mg) were dissolved in phosphate-buffered saline and treated with collagenase. They were transferred to centrifuge tubes, centrifuged at 10,000 rpm for 10 minutes, and photographed. a: NCol, b: CCol-1, c: CCol-2, d: CCol-3. Figure S2: FT-IR spectra of natural collagen (NCol) and L-Glu-cross-linked collagen (CCol series). Figure S3: Live/dead fluorescence images of osteoblasts cultured on collagen scaffolds for 72 h: control (cells only), non-cross-linked collagen (NCol), and L-Glu-cross-linked collagen scaffolds (CCol-1, CCol-2, and CCol-3). Live cells were stained green with calcein-AM and dead cells were stained red with ethidium homodimer I.
