# Supplementary material for: Amine-Selective Crosslinking of Collagen via Pre-Activated L-Glutamic Acid for Maintaining Ionic Interactions and Enhancing Mechanical and Biological Performance
Source: Polymers (Basel). 2026 Jul 20;18(14):1766. doi: 10.3390/polym18141766 (PMC13418526; doi:10.3390/polym18141766)
Supplement: Supplementary file 1 [file polymers-18-01766-s001.zip › polymers-4379543-supplementary.pdf]

Supplementary Information:

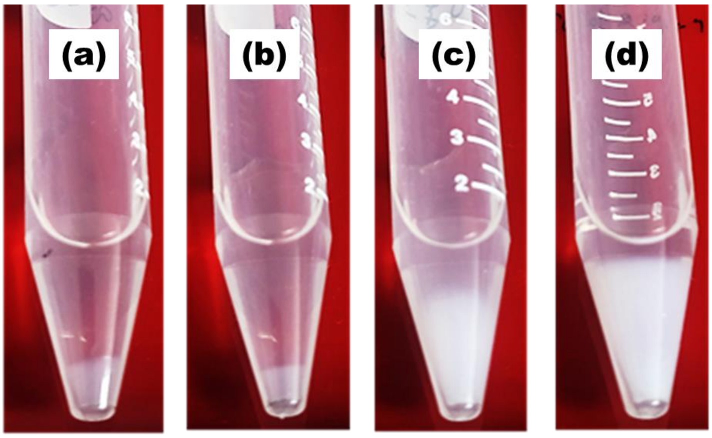

Fig. S1 Non-cross-linked collagen and cross-linked collagen (70 mg) were dissolved in phosphate-buffered saline and treated with collagenase. They were transferred to centrifuge tubes, centrifuged at 10,000 rpm for 10 minutes, and photographed. a: NCol, b: CCol-1, c: CCol-2, d: CCol-3.

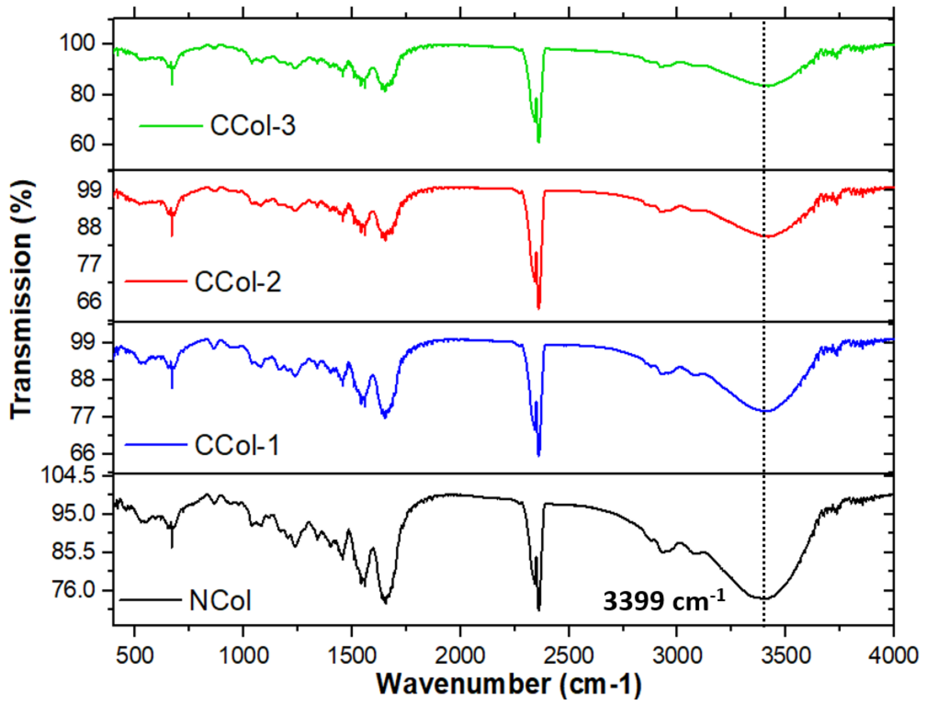

Fig. S2 FT-IR spectra of natural collagen (NCol) and L-Glu-cross-linked collagen (CCol series).

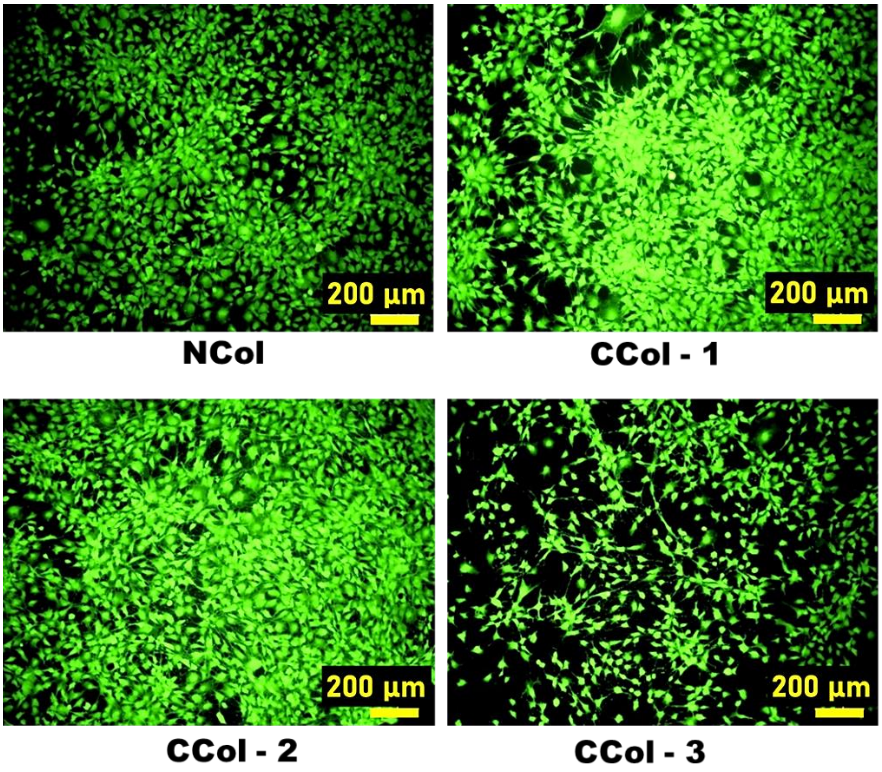

Fig. S3 Live/dead fluorescence images of osteoblasts cultured on collagen scaffolds for 72 h: control (cells only), non-cross-linked collagen (NCol), and L-Glu-cross-linked collagen scaffolds (CCol-1, CCol-2, and CCol-3). Live cells were stained green with calcein-AM and dead cells were stained red with ethidium homodimer I.
